# Catheterisation laboratory is the place for rehabilitating the pulmonary arteries

**DOI:** 10.4103/0974-2069.43875

**Published:** 2008

**Authors:** Bhava RJ Kannan, Shakeel A Qureshi

**Affiliations:** Department of Pediatric Cardiology, Evelina Children's Hospital, London, UK

**Figure 1:**
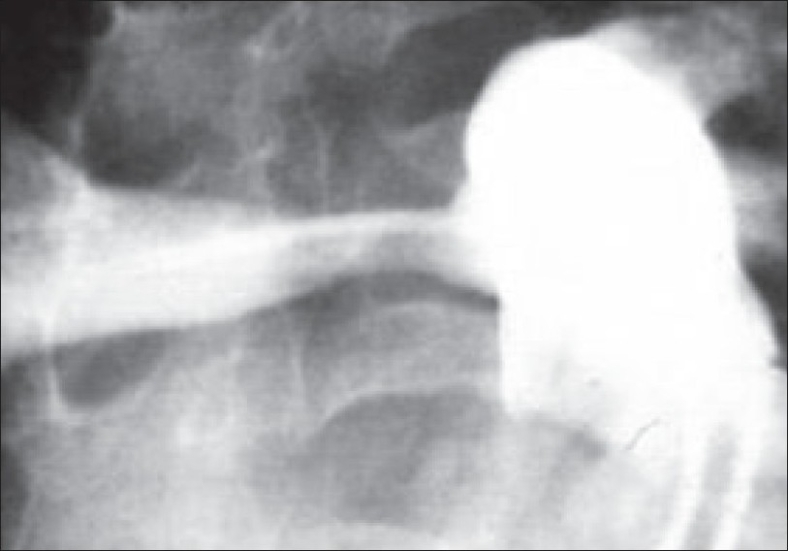
Pulmonary artery angiogram in right anterior oblique projection showing a congenital localised stenosis at the origin of the right pulmonary artery with post-stenotic dilatation

**Figure 2:**
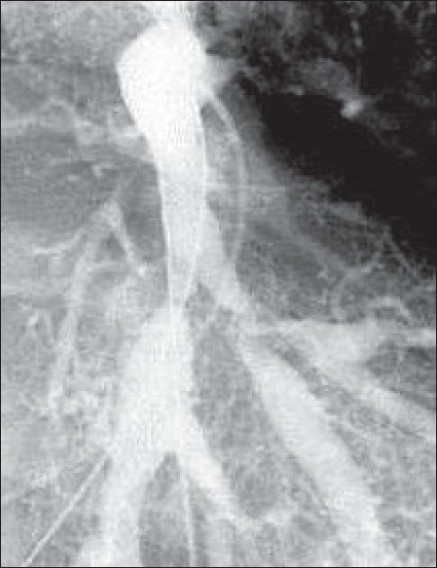
Pulmonary artery angiogram in antero-posterior projection showing congenital multiple distal left pulmonary artery stenoses beyond the hilum

**Figure 3:**
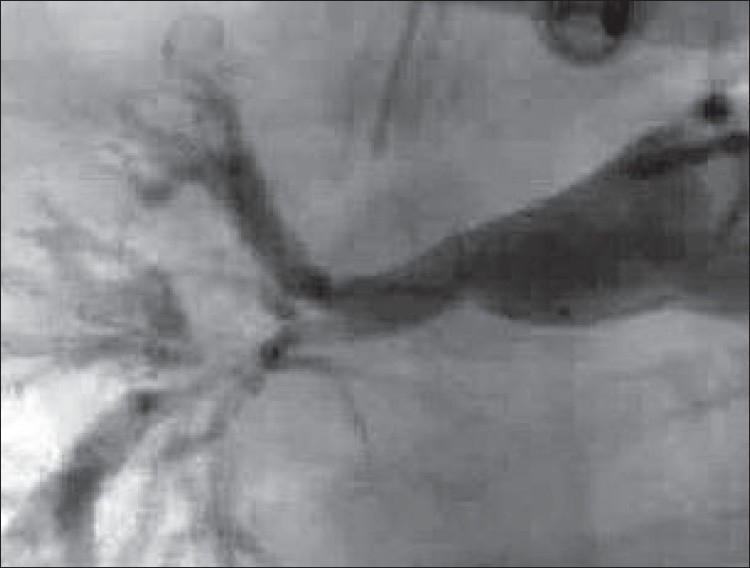
Pulmonary artery angiogram showing muliple right pulmonary artery stenoses in a patient with tetralogy of Fallot

Pulmonary artery stenosis exists as a spectrum. It varies from localized stenosis occurring in one or both branches at one end of the spectrum to more diffuse widespread bilateral stenoses at the other. Very often a localized stenosis is associated with post-stenotic dilatation [Figure 1] whereas diffuse tubular narrowing may not have post-stenotic dilatation. The stenosis can occur either at the origin or more peripherally beyond the hilar branches [Figure 2]. The stenosis is commonly congenital in origin occuring either as an isolated anomaly or in association with other congenital heart defects. It can also occur following a previous attempt at surgical repair. It is most frequently seen in children with pulmonary atresia with ventricular septal defect or more complex anomalies with single ventricle physiology [Figure 3]. In pulmonary atresia with ventricular septal defect, about 20% of patients have stenosis of the left pulmonary artery and approximately 10% have stenosis involving the origin of the right pulmonary artery.[1] In an angiographic study, 70% of cases of pulmonary atresia and 10% of cases of tetralogy of Fallot had juxtaductal pulmonary artery stenosis.[2] In another study of 25 neonates with congenital heart disease, branch pulmonary artery stenosis was found in 36% of patients[3] occurring in both biventricular and univentricular hearts. Extension of the ductal tissue into the pulmonary artery is probably responsible for the branch pulmonary artery origin stenosis and hence it is also referred to as pulmonary artery coarctation. Congenital bilateral branch pulmonary artery stenoses is known to occur with various conditions such as rubella, Alagille syndrome, cutaneous laxa, Noonan's syndrome, Ehlers-Danlos syndrome and Williams syndrome. Acquired branch pulmonary artery stenosis occurs after previous surgical attempt at repairing the stenosis. There is an increased risk for development of post-operative pulmonary artery stenosis in children with hypoplastic branch pulmonary arteries, after duct ligation, after insertion of a Blalock-Taussig shunt, after placement of a pulmonary artery band, after pulmonary arteriotomy and following anastomosis of any kind such as those after an arterial switch operation or implantation of a right ventricle to pulmonary artery conduit.

Some children with stenosis of a branch pulmonary artery are at a risk of developing ipsilateral pulmonary artery hypoplasia[4] and are predisposed to developing spontaneous occlusion and disconnection from the main pulmonary artery.[5] Hence it is essential to manage pulmonary artery stenosis effectively. It is important to establish equal and unobstructed blood flow into both pulmonary arteries as early as possible in all the children who would need two ventricle repair and more importantly, in those who have been committed to a Fontan operation in future.

The indications for intervention (surgical or transcatheter) include right ventricular systolic pressure greater than 50% of systemic pressure in the presence of bilateral branch pulmonary artery stenoses, or lung perfusion to the ipsilateral lung of less than 20% in the presence of unilateral stenosis. If the patient has pulmonary regurgitation or syndromes such as Alagille syndrome, then the indications for intervening are less rigid. The aims of treatment are to balance the pulmonary circulation equally to the two lungs in order to improve the pulmonary function, to reduce right ventricular hypertension and to increase the blood flow to the distal pulmonary arteries thereby reducing the degree of pulmonary regurgitation. The treatment of branch pulmonary artery stenosis may be needed prior to corrective surgery, during or after corrective surgery or as an independent palliative measure.

Branch pulmonary artery stenosis is dealt with either surgically or by transcatheter interventional techniques. Surgical reconstruction of pulmonary arteries is technically very demanding. Various surgical techniques have been described to manage these stenoses. They include pericardial patch augmentation, pericardial pedicle graft and Gore-Tex patch augmentation. Kim *et al*, showed that pulmonary angioplasty and placing a Blalock-Taussing shunt at the site of pulmonary artery coarcation resulted in a balanced increase in the pulmonary artery index on both sides.[6] Kaneko *et al*, introduced ‘lay open technique’ of pulmonary arterioplasty and showed an increase in the pulmonary artery diameter commensurate with somatic growth without any significant aneurysm formation or restenosis during a relatively short mean follow up of 1.9 years in a small cohort of patients.[7] Isolated case reports are available on successful staged surgical reconstruction of severe bilateral branch pulmonary stenoses.[8] Various reports have emphasized the need for complete excision of the ‘ductal’ tissue from the branch pulmonary artery for the prevention of restenosis.

Despite these reports of good short term results following surgery, there is a high rate of restenosis of the branch pulmonary arteries requiring repeat surgery in these children.[9,10] Most of these children remain asymptomatic as the restenosis occurs gradually allowing for the compensation by the contralateral pulmonary artery and hence, the restenosis may often go unrecognised. As a result, surgical series tend to underreport this complication if the follow up is based only on symptoms. Sabiniewicz *et al* performed lung perfusion scintigraphy in 87 patients following surgical repair of tetralogy of Fallot and found abnormal perfusion in 49%, mostly involving the left lung.[11] Angiography revealed stenosis of branch pulmonary arteries in 35% of the patients with an abnormal perfusion scan and in 9% with a normal lung perfusion scan.[11] Petit *et al*, performed cardiac cathetererisation in 33 patients following complete repair of tetralogy of Fallot with branch pulmonary artery stenosis. At a mean follow up of 2.5 years, only one third of the patients had a normal pulmonary arterial tree.[9] In a small study comprising of 15 infants with pulmonary coarctation who underwent surgical reconstruction, there was an increase in the pulmonary artery size on both sides. However, repeat surgery was needed in 5 out of these 15 patients.[8] On some occasions, repaired branch pulmonary arteries get occluded and disconnected from the main pulmonary artery.[5,10] In such cases, it is very difficult to perform any transcatheter intervention.

From this data, it is clear that although surgeons can repair origin stenosis of the branch pulmonary arteries, results are far from satisfactory. Thus, dealing with branch pulmonary artery stenosis by surgery is not an optimum long term option. Furthermore, surgical access is limited upto the level of the hilum and any stenosis of the pulmonary artery beyond the hilum can not be dealt with surgically. The alternative treatment is by transcatheter techniques. Most of the literature on transcatheter management of branch pulmonary artery stenosis is in the post-operative patients. The transcatheter interventional options consist of conventional or high pressure balloon angioplasty, cutting balloon angioplasty and stent implantation.

Perry and colleagues reported a low success rate and significant complications with balloon angioplasty using low pressure balloons in late 80s.[12] Successful dilatation was achieved in only 50% of cases and the use of oversized balloons resulted in complications such as reperfusion pulmonary edema, rupture of the pulmonary arteries and death in some cases. With the introduction of high pressure balloon, the results improved significantly.[13] However, development of pulmonary artery aneurysm was seen as a late complication. With the advent of cutting balloon, dilatation of severely resistant peripheral pulmonary artery stenosis was attempted with acceptable results.[14] Cutting balloons have been shown to be successful in all the subsets of patients including pulmonary artery stenosis associated with tetralogy of Fallot, Williams syndrome and Allagille syndrome which are resistant to routine balloon angioplasty.[15] Cutting balloon dilatation results in a more controlled dissection of the intima with angiography showing evidence of vascular damage. Although, this dissection is well tolerated in the majority, some patients require placement of intravascular stents. Bergersen *et al*, showed that the initial gain in the luminal diameter after use of cutting balloons is usually maintained during the follow up despite the vascular trauma induced by these balloons.[16]

Due to unpredictable and unsatisfactory long term results following surgery and plain balloon angioplasty, stents have been used more commonly with a success rate reaching upto 90%. In a large series involving implantation of pulmonary artery stents in 338 patients over the last two decades, McMahon *et al*, reported absence of procedure related morbidity or mortality since 1997.[17] There are numerous reports of successful stenting of the branch pulmonoary artery stenosis in the post operative patients.[18–23] Although stents are commonly placed after balloon dilatation, in recent times, direct stenting is performed especially for long segment stenosis, in which results with plain balloon angioplasty are unsatisfactory.[21] Whenever the surgery involves reconstruction of the right ventricular outflow tract, the pulmonary artery anatomy may get distorted with aneurysmal dilation. This results in difficulty in cannulating the branch pulmonary artery at cardiac catheterization. In such cases, a redo-sternotomy or thoracotomy is needed for direct access to the branch pulmonary artery.[23]

Indications and use of stents for dealing with pulmonary artery stenosis have increased in the recent times. Stents are mainly used to deal with origin stenosis occurring naturally or after previous surgery, kinking or tenting of the branch pulmonary artery (usually seen after previous surgery), external compression of the branch pulmonary artery, elastic recoil (rendering an attempt at balloon dilation futile), an intimal tear after balloon angioplasty and recanalization of totally occluded vessel.

**Figure 4:**
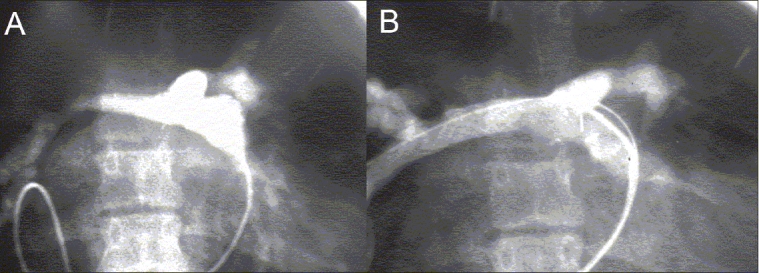
(A) A combination of shunt-related and congenital right pulmonary artery stenosis in a patient with tetralogy of Fallot, (B) The angiographic result after implantation of two overlapping Palmaz stents prior to surgical correction

**Figure 5:**
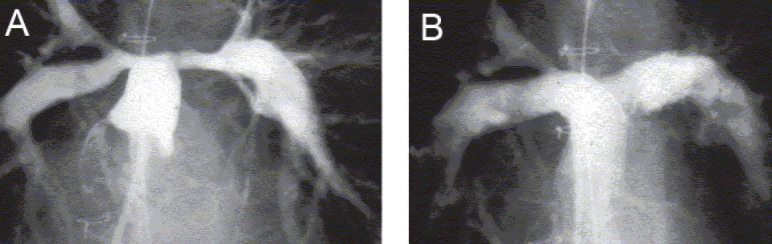
(A) A still-frame angiogram showing bilateral origin stenoses of pulmonary arterial branches after previous surgery for tetralogy of Fallot, (B) The angiographic result after simultaneous implantation of stents at the origins of the two branches

**Figure 6:**
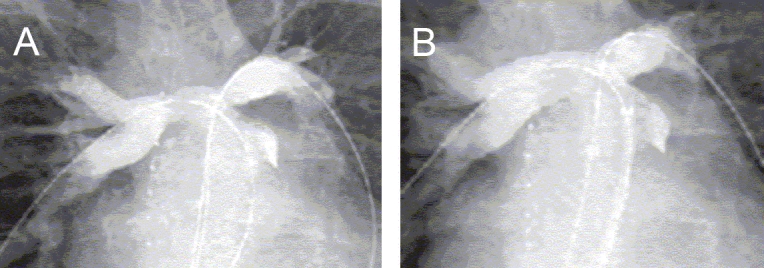
(A) A still-frame angiogram showing bilateral origin stenoses of pulmonary artery branches in another patient after previous surgery for pulmonary atresia with ventricular septal defect, (B) The angiographic result after implantation of stents at the origins of the two branches

**Figure 7:**
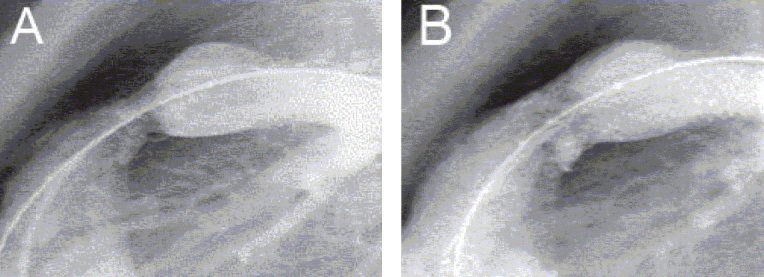
(A) A stenosis within the right ventricle to pulmonary artery conduit in a patient who had previously undergone surgical repair of common arterial trunk. (B) The angiographic result after stenting of the conduit. The resulting reduction in the right ventricular hypertension allowed a delay of several years before replacement of the conduit was needed.

**Figure 8:**
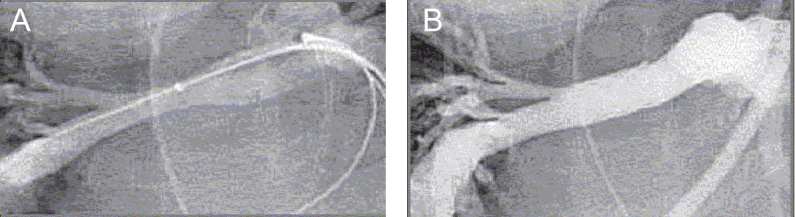
(A) A right pulmonary artery angiogram in the right anterior oblique projection showing significant stenosis in its proximal portion, (B) The angiographic result after implantation of a Palmaz stent in the right pulmonary artery

**Figure 9:**
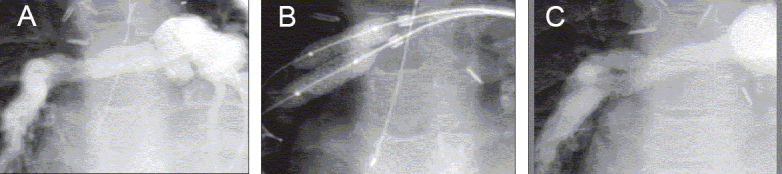
(A) Stenosis in adjacent branches of the right pulmonary artery beyond the hilum in a patient who had undergone staged treatment of pulmonary atresia, ventricular septal defect and recruitment of major aortopulmonary collateral arteries. These stenoses were from the recruited collaterals. (B) Simultaneous implantation of two Palmaz premounted stents. (C) An excellent angiographic result of stent implantation

Stenting may be performed to rehabilitate branch pulmonary artery stenosis pre-operatively, for example in patients with distal pulmonary artery stenosis in association with tetralogy of Fallot [Figure 4 A and B]. In patients who have undergone repair of pulmonary atresia with ventricular septal defect, or arterial switch operation, in whom surgery involved reconstruction of the origins of the pulmonary arteries, bilateral branch stenoses may recur and this can be effectively treated by simultaneous implantation of stents in both the branches [Figure 5 A and B, Figure 6 A and B]. In infants, stenting is used as a bridge to delay conduit replacement. One example of this is pulmonary artery stenosis occurring early after correction of common arterial trunk. In these children, stenting of the conduit or the branches of the pulmonary artery may delay the timing of the replacement of the conduit [Figure 7 A and B, Figure 8 A and B]. Implanting stents in such small infants, although technically challenging, can produce gratifying results. Some stents, such as the Palmaz Genesis stent, can be dilated serially up to 18 mm diameter and does not need removal. Some patients with complex stenoses involving the adjacent branches can also be stented effectively [Figure 9 A–C].

If the baby is too small (weight less than 5 kg) at the time of intervention, in recent years, a hybrid approach is used for intra-operative or preoperative stenting of branch pulmonary artery stenosis. Ungerleider *et al* have reported their experience in 22 patients, in whom stents were placed under direct vision in the operating theatre on cardiopulmonary bypass.[24] The procedure was successful in all patients resulting in a significant reduction in the post-operative gradients. This approach avoids difficult surgery on fragile pulmonary arteries, recruits pulmonary arteries, maintains their patency and makes them amenable to future percutaneous or surgical interventions.[20,25] In severe forms of pulmonary arterial coarctation, the pulmonary arteries may be non-confluent or discontinuous. In these situations, interventional therapy does not have a primary role. However, in one report, temporary revascularization of disconnected left pulmonary artery was achieved by transcatheter recanalisation of the closed ductus arteriosus, which facilitated the later surgical treatment.[26]

**Figure 10:**
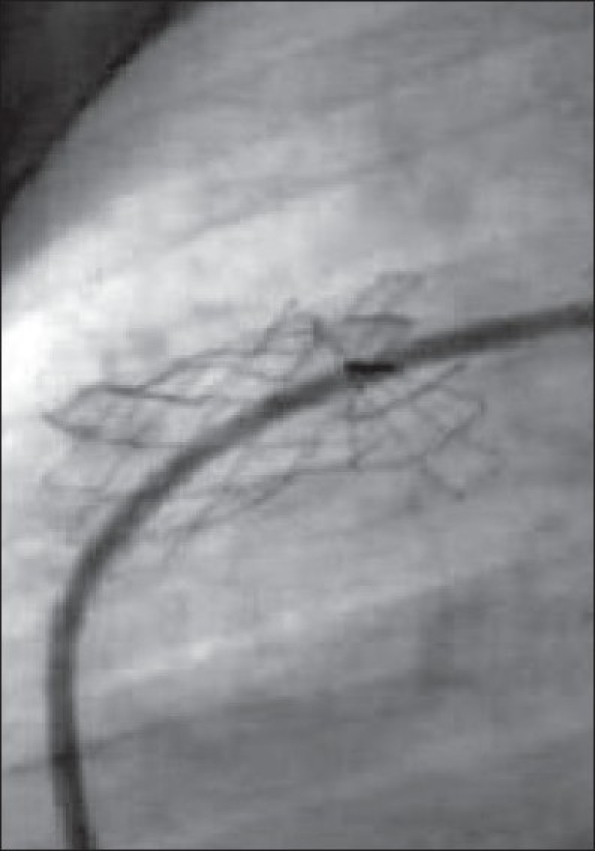
Stent fracture in a patient in whom a Palmaz stent was implanted in a right ventricle to pulmonary artery conduit

Although stenting is very effective and reasonably safe, it has its own share of complications. These include stent malposition, stent migration, jailing of side branches, stent fracture [Figure 10], dissection of the vessel and vessel rupture. In a multicentre study looking at the untoward effects of stenting in patients with all forms of congenital heart disease, the overall complication rate was 19% of which 6% were classified as “major” including 2.3% procedure-related deaths. Stent malposition and embolisation were the commonest complications.[27,28] These can be significantly reduced with the use of premounted stents, but these are not always available in appropriate sizes and lengths. Two more concerns related to stent implantation in branch pulmonary arteries are the need for redilatation in a growing child and the feasibility of surgical procedure on the stented pulmonary arteries. It is reassuring that various authors have reported the efficacy and safety of stent redilatation even 10 years after the initial procedure.[29–31] The main reason for the redilatation was to adapt the stent diameter to the growth of the patient. Other reasons that warranted redilatation were: external compression, intentional staged serial dilatation to avoid vessel overdilatation during the initial stent implant and neointimal proliferation resulting in “in-stent restenosis”. However, the restenosis rate has been reported to be low, ranging between 2-3%. Repeat dilatations allow further growth in the vessel distal to the stent by increasing the blood flow. Overdilatation of stents at the time of primary implantation is considered to accelerate neointimal proliferation and should be avoided. The presence of a stent in the pulmonary artery branch origin does not seem to interfere with the suture line during subsequent surgery.[24,32] Incising across the stents and suturing through their interstices, most often, do not present a significant problem to the surgeons. There are reports of successful Fontan completion in patients with previously stented pulmonary arteries.[20]

**Figure 11:**
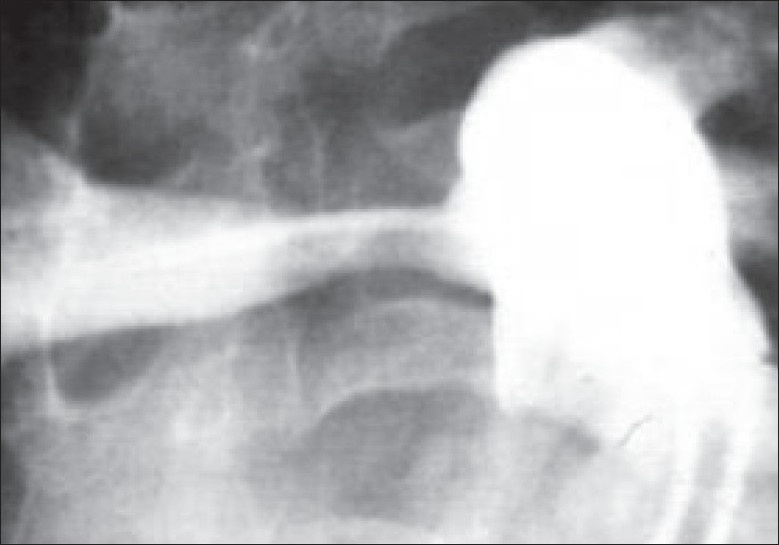
An angiogram of the same patient as in figure 1 after implantation of a Palmaz stent at the origin of the right pulmonary artery showing excellent result

In conclusion, with continuing developments and refinements in the technology and increasing experience with these techniques, the safety profile of stenting of branch pulmonary arteries should improve even further. Although surgeons can deal with proximal branch pulmonary artery stenosis, there is a high probability of recurrence. Moreover, surgeons are unable to deal with stenoses beyond the hilum. Stents can be implanted at all ages with excellent and predictable results [Figure 11], but in children, the stents will need redilation to keep pace with growth of the child. Nevertheless, this is probably more acceptable than a repeat operation. There are occasions when a hybrid approach may be needed to stent the branch pulmonary arteries. Thus, based on the available data and our own experience, we strongly believe that catheterization laboratory (and not the operating room) is the ideal place for dealing with majority of pulmonary artery stenosis.
